# A Learning Health Care System Using Computer-Aided Diagnosis

**DOI:** 10.2196/jmir.6663

**Published:** 2017-03-08

**Authors:** Amos Cahan, James J Cimino

**Affiliations:** ^1^ IBM TJ Watson Research Center Yorktown Heights, NY United States; ^2^ Informatics Institute University of Alabama at Birmingham Birmingham, AL United States

**Keywords:** diagnostic errors, diagnosis, computer-assisted, decision support systems, clinical, pattern recognition, automated, knowledge bases, knowledge management, diagnosis support systems, crowdsourcing, structured knowledge representation

## Abstract

Physicians intuitively apply pattern recognition when evaluating a patient. Rational diagnosis making requires that clinical patterns be put in the context of disease prior probability, yet physicians often exhibit flawed probabilistic reasoning. Difficulties in making a diagnosis are reflected in the high rates of deadly and costly diagnostic errors. Introduced 6 decades ago, computerized diagnosis support systems are still not widely used by internists. These systems cannot efficiently recognize patterns and are unable to consider the base rate of potential diagnoses. We review the limitations of current computer-aided diagnosis support systems. We then portray future diagnosis support systems and provide a conceptual framework for their development. We argue for capturing physician knowledge using a novel knowledge representation model of the clinical picture. This model (based on structured patient presentation patterns) holds not only symptoms and signs but also their temporal and semantic interrelations. We call for the collection of crowdsourced, automatically deidentified, structured patient patterns as means to support distributed knowledge accumulation and maintenance. In this approach, each structured patient pattern adds to a self-growing and -maintaining knowledge base, sharing the experience of physicians worldwide. Besides supporting diagnosis by relating the symptoms and signs with the final diagnosis recorded, the collective pattern map can also provide disease base-rate estimates and real-time surveillance for early detection of outbreaks. We explain how health care in resource-limited settings can benefit from using this approach and how it can be applied to provide feedback-rich medical education for both students and practitioners.

## Why We Need Computer-Aided Diagnosis

### We Make Too Many Diagnostic Errors

Two main questions are key when evaluating a patient in the context of constructing a differential diagnosis: The first is “How representative is the presentation of the patient to a set of manifestations of a known disease?” In other words, to what degree is there a match between a set of symptoms, signs, and laboratory results and the clinical features of the disease. The second is “What is the likelihood of encountering that disease in a patient like this?” Answering this question requires knowing the base rate (ie, incidence) of the disease and accounting for any patient risk factors that may alter the patient’s prior probability of having the disease.

Good clinicians are characterized by their ability to cluster findings around a single process or cause. Their intuitive clinical assessment heavily relies on pattern recognition [1]. It has been argued [2] that, to develop skilled intuition, a predictable environment and adequate opportunity to practice skills are needed. Indeed, physicians are more likely to be wrong in cases where they have encountered too few instances of a pattern to recognize it.

However, a perfect match between a patient’s presentation and a typical clinical picture of a disease is no guarantee that the patient indeed has that disease. The aphorism coined by Dr Theodore Woodward, “When you hear hoofbeats, think of horses not zebras” [3], reflects the importance of the disease’s prior probability in the population to which the patient belongs. In other words, an atypical presentation of a common disease is probably more likely to be encountered than a classic presentation of a rare disease.

Although probabilistic reasoning is key to medical diagnosis, physicians, like humans in general, perform poorly in this aspect; probability overestimation and low between-physician agreement are common [4-6]. The US National Academy of Medicine’s recently published *Improving Diagnosis in Health Care* report [7] points to the unacceptable number of patients harmed by diagnostic errors. With medical knowledge rapidly expanding, information lacunae are common [8]. Doctors use heuristics (mental shortcuts) to compensate for knowledge gaps, but this practice involves substantial biases [9,10], which may contribute to diagnostic errors. Thus, to minimize diagnostic errors, doctors need help.

Attempts to develop efficient computer-aided diagnosis support systems (DSSs) [11,12], including differential diagnosis generators, have been made since the 1960s (reviewed in [13,14]). Whereas narrow spectrum, rule-based systems, such as electrocardiogram interpreters, have become ubiquitous over the years, DSSs in the general or internal medicine domain have not [15]. This, despite a demonstrated positive effect on physician performance [16]. Several general medicine DSSs are available commercially (eg, DXplain [17], GIDEON [18], and Isabel [19]), but their routine clinical use remains limited. In fact, only 5 of 11 differential diagnosis generators included in a recent systematic review [20] are currently used in practice. Despite the digital revolution of the health care system in the last decade, a 2013 review of DSSs concluded that progress in the development of DSSs during this time was minimal [21]. However, recent years have seen a new class of computerized diagnosis tools aimed at patients. These resources, called “symptom checkers,” suggest potential diagnoses explaining a user’s set of symptoms as reported through a user interface [22]. A recent evaluation of 23 symptom checkers [23] using standardized cases found that the correct diagnosis was listed among the top 20 options in 58% (95% CI 55%-62%) and appeared first on the list in 34% (95% CI 31%-37%). Symptom checkers provided inappropriate triage advice in 20% of emergent and 45% of nonemergent cases.

### Why Diagnosis Support Systems Fail

Poor specificity of DSSs is reflected by the large number of possible diagnoses suggested. Berner and coworkers [24], in a report on leading general DSSs, used “relevance,” a measure closely related to specificity. They defined relevance as the average proportion of computer-generated diagnoses considered reasonable by clinical experts. Mean relevance scores were low (ranging from 0.19 to 0.37). Poor relevance rates mean that a workup plan based on DSS-suggested diagnoses would be impractical and would expose patients to undue risks [25].

Indeed, by design, DSSs focus primarily on sensitivity at the expense of specificity [26], yet in Berner and colleague’s report, the overall sensitivity (the proportion of cases where the correct diagnosis was included in the computer-generated differential diagnosis list) ranged from 0.52 to 0.71. When they considered only the top 5 and 10 diagnoses on the list, rates were only about 0.25-0.35 for relevance and 0.35-0.45 for sensitivity. A follow-up study found similar results in a different set of DSSs [26]. In fact, a Google Web search has been reported to provide the correct diagnosis at similar rates [27].

**Figure 1:**
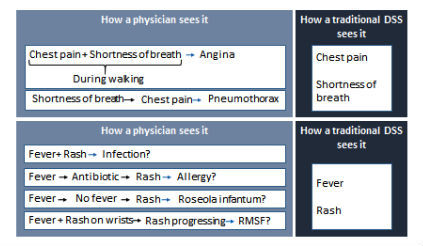
Examples of the view of a set of findings in a patient by a physician and a traditional diagnosis support system (DSS): chest pain and shortness of breath (upper panel), fever and rash (lower panel). Temporal and semantic interrelations between findings are crucial in putting findings in the right clinical context. RMSF, Rocky mountain spotted fever.

Current DSSs cannot efficiently match patients and diseases on patterns, since they rely on a unidimensional projection of clinical information; typically, the system uses a vector of “findings” (symptoms, signs and laboratory results) provided by the user to generate a differential diagnosis. Some systems differentiate between acute and more prolonged processes [17,18], but none are able to cluster findings based on their course in time. Using this “bag of findings” approach makes DSSs agnostic to key clinical clues. For instance, chest pain and dyspnea appearing during physical exercise strongly suggest angina, whereas shortness of breath with subsequent chest pain may suggest pneumothorax or a pulmonary infarction. From a DSS point of view, these conditions are indistinguishable, as both have the same findings: “chest pain” and “dyspnea” (Figure 1). Indeed, some systems define higher-level concepts such as “angina chest pain” [28], but these are rigid and limited in number.

An incomplete knowledge base further limits DSS performance. INTERNIST-1 included 570 diseases [29], and DXplain’s knowledge base has over 2400 diseases and 5000 findings [30]. Nevertheless, when 4 DSSs were evaluated on a set of challenging clinical cases, the correct diagnosis was absent from their knowledge base in 9% to 27% of cases [24]. Manually curating and maintaining a comprehensive knowledge base in the face of rapid knowledge growth is extremely demanding. In fact, leading informaticians have acknowledged that even working toward a complete DSS knowledge base is infeasible [15]. In a seminal paper published in 1959 [31], Ledley and Lusted proposed a physician-maintained, notched card-based “learning device.” This device could be used to collect and reuse associations between symptoms and diseases, with each card representing a patient. This early attempt to capture physician knowledge as applied to a single patient in a structured, machine-interpretable format allowed, in theory, for supporting a learning DSS. An attempt to implement knowledge accumulation through actual cases was made in the 1980s by the creators of the discontinued ILIAD expert system [28]. More recently, the Web-based Human Diagnosis Project [32] was introduced, which allows clinicians to upload real or simulated case vignettes, and challenges other clinicians to solve those cases. This information is used to generate a knowledge graph associating symptoms, signs, and diagnoses. Nevertheless, to the best of our knowledge, there is no commercially available DSS that has self-learning capabilities.

Almost 60 years after Ledley and Lusted [31] had laid the foundations for probabilistic reasoning in diagnosis making (with ILIAD being an exception to some degree), DSSs still do not hold disease base rates as part of their knowledge bases and are unable to account for factors that may alter the prior probability of a disease. Some diseases are limited to certain geographic locations, while the incidence of others varies by the time of year or by race. Readily available in the digital age, these data remain unused by current DSSs to refine their differential diagnosis.

Finally, DSSs do not align well with clinicians’ work flow. A few DSSs now offer variable degrees of direct connectivity to the electronic health record (EHR) [33]. Some can extract data from the EHR using natural language processing tools, although this may adversely affect performance [34].

## Next-Generation Diagnosis Support Systems

Reviewing DSSs in 1994 [13], Miller noted that

We may understand, in theory, how to develop systems that take into account gradations of symptoms, the degrees of uncertainty...the severity of each illness under consideration, the pathophysiologic mechanisms of disease, and/or the time courses of illness. However, it is not yet practical to build such broad-based systems for patient care.

More recently, Weber et al note that “industries have figured out...that big data becomes transformative when disparate data sets can be linked at the individual person level” [35]. Technology is now ripe to enable the development of next-generation DSSs (NGDSSs) based on these key insights.

**Figure 2:**
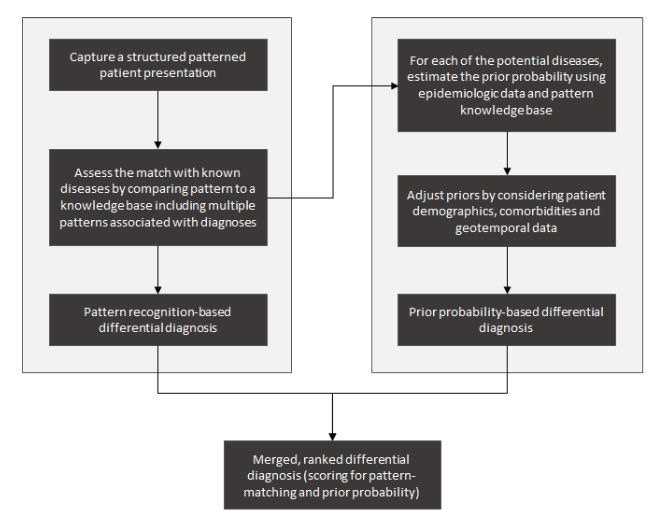
An integrated approach to computer-aided diagnosis. The process addresses the 2 questions that lead to likely diagnoses: Left side: How similar is the presentation of the patient to a set of manifestations of a known disease? Right side: What is the likelihood of encountering that disease in a patient like this?

Here we portray NGDSSs and provide a conceptual framework for their development (Figure 2). To be effective, NGDSSs will have to (1) support pattern recognition-based diagnosis by capturing a richer clinical picture, (2) provide personalized prior-probability assessments, (3) maintain a comprehensive and current knowledge base, and (4) better align with clinicians’ workflow. In the next sections, we discuss NGDSSs characteristics.

### Structured Patterned Patient Presentation

For hundreds of years, physicians have been documenting their thoughts in the form of free text. Computer systems work best with structured data, but free-text input prevails even in the age of EHRs. A digital form of Ledley and Lusted’s learning device could provide a structured representation of a patient’s symptoms, extracted using natural language processing, as they relate to a disease. However, such a representation would, again, be of the “bag of findings” kind, as natural language processing techniques cannot reliably generate structured representations of complex clinical concepts documented in the EHR notes. In particular, this is true for temporal and semantic interrelations between symptoms and signs, which are key in forming the clinical patterns recognized by physicians. Thus, new ways to provide DSSs with a structured clinical picture are needed.

We suggest that a structured, higher cognitive-level patient representation can be constructed in real time through a (graphical) machine-physician interaction. We refer to this representation as a “structured presentation pattern” or “structured pattern.” A structured pattern can be thought of as a model, which can represent physician knowledge and reasoning in a machine-interpretable format. A structured pattern should ideally represent key symptoms and signs associated with a particular patient’s presentation and their temporal and semantic interrelations. This allows for translation of a list of findings (symptoms and signs) into multiple distinct structured patterns according to the temporal course of the disease and other relations between findings. Through this approach, a differential diagnosis constructed by NGDSSs is likely to be more specific than one based on a list of findings.

#### Temporal Patterns

The creators of the pioneering INTERNIST-1 attributed its insufficient clinical reliability in part to it’s being temporally naive [36]. Kohane [37], appreciating the importance of temporal patterns in clinical reasoning, concluded that “knowledge bases that fail to capture the temporal component of the course of disease omit useful diagnostic knowledge.” It is hard to estimate the percentage of cases in which the course in time of the manifestations of a disease plays a substantial role in diagnosis making. Yet the fundamental categorization in clinical medicine of illnesses as acute, subacute, and chronic attests to the central role of the temporal dimension in differentiating between diseases. Still, efforts to capture, model, and represent temporal relations between clinical entities [37-40] have not yet matured to support temporally aware DSSs.

#### Semantic Patterns

One piece of evidence can be the cause of another, one may support or contradict the other, or one may be more reliable than another. Interpretation of a symptom or sign is ever dependent on the clinical context, which is, in a sense, a sum of all such interrelations. For example, a patient with suspected brucellosis may be unsure of having consumed potentially unpasteurized milk products in the preceding weeks. A physician auscultating the heart may hear an extra sound during diastole but have doubts as to whether this is an opening snap or a third heart sound. A patient’s record may document contradicting views of the etiology of a prior illness (eg, convulsion vs transient ischemic attack). Making at least some of these semantic or contextual interrelations interpretable by NGDSSs is likely to improve their performance on pattern recognition.

### Using Big Data to Create a Differential Diagnosis

Patient notes include protected health information, which is why individual medical records cannot be readily shared. Free text can only be considered to be deidentified after it has been manually reviewed. In contrast, user-generated structured patient patterns are readily automatically de-deidentifiable.

This opens the way to *real-time* sharing of high-quality deidentified clinical patient information across physicians and institutions. The collective experience of physicians worldwide can be stored in a structured knowledge base made available to support pattern recognition-based diagnosis. Similarity analysis could support this process by computing the degree of match between a patient’s pattern and patterns of other patients in the knowledge base who already have a diagnosis. This can provide an answer to the first diagnostic question mentioned above, namely, “How similar is the presentation of the patient to a set of manifestations of a known disease?”

Disease prevalence by parameters such as age, sex, race, and geotemporal distribution can be extracted from various sources, including published reports, large EHR repositories, administrative claims data, social media, and environmental data (eg, weather). These sources can feed an NGDSS knowledge base. Patient demographic data automatically extracted from the EHR can personalize prior-probability estimates. Few findings typical of a particular disease are invariably present in every case of it. The probability of a certain symptom occurring, a certain sign being noted, or a particular laboratory abnormality being found in a given disease is available from published reports [41] and can be used by NGDSSs. In fact, as the NGDSS knowledge base expands, the collective body of structured patterns contributed by physicians can serve as a living epidemiologic database, providing real-time statistics on the incidence of symptoms, signs, and diseases. This will enable NGDSSs to address the second diagnostic question: “What is the likelihood of encountering that disease in a patient like this?”

### A Democratized Medical Knowledge Base

DSSs partly rely on the fact that disease manifestations change relatively little over time, yet as new diseases arise (with obvious examples being human immunodeficiency virus and Zika virus infections) and new disease correlates are found (eg, genetic traits), continuous updates are necessary [13].

**Figure 3:**
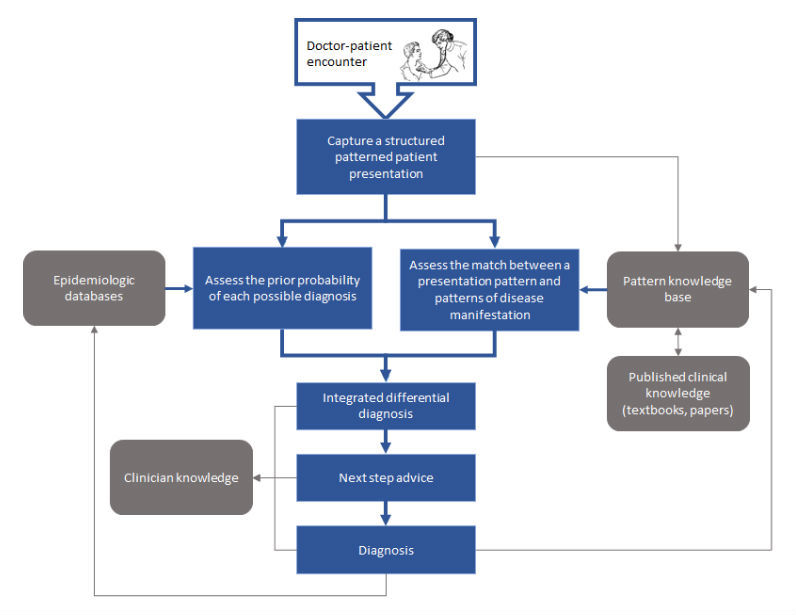
Generating a real-time structured representation of a patient presentation supports a computer-aided diagnostic process (blue arrows) and a learning health care system through knowledge reuse (gray arrows).

Using structured patterns, crowdsourcing of knowledge collection and reuse becomes possible. Crowdsourcing may be a sustainable strategy in a reality of exploding knowledge and limited resources (Figure 3).

**Figure 4:**
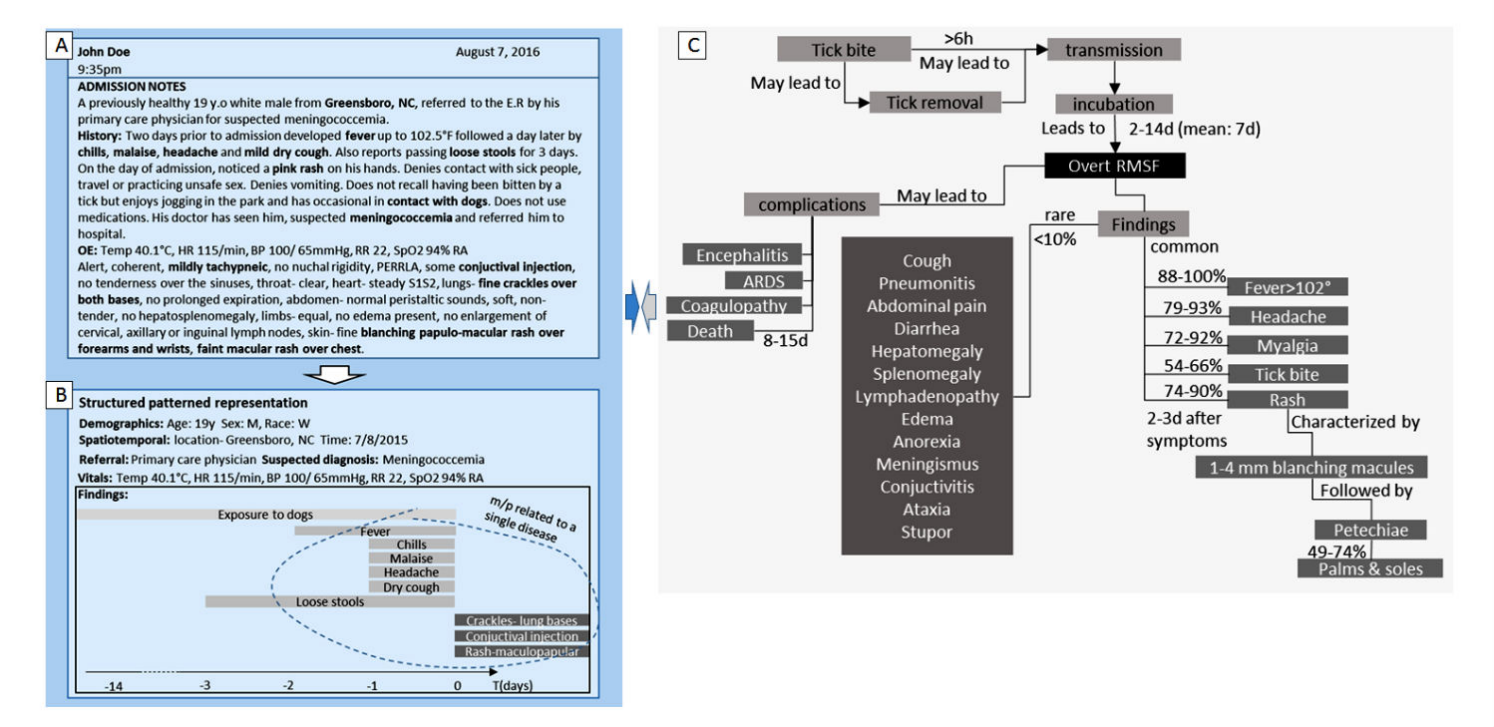
Structured patient and disease representation. (A) A simulated view of an electronic health record with admission notes. Key terms are highlighted automatically using a real-time natural language processing engine or marked by the user. (B) Selected terms are then manipulated by the user by means of a touch screen to create a pattern representing key temporal and semantic interrelations between terms in a structured format. This pattern is augmented by automatically extracted relevant clinical data, demographics, and other metadata. (C) Structured patterned representation of the manifestations of Rocky Mountain spotted fever derived from a review article. Applying analytics schemes for assessing patient and disease similarity in context of disease prevalence can inform the generation of a ranked differential diagnosis for the patient in question.

Each time a physician adds a patient pattern (subsequently labeled with a diagnosis code assigned to that patient), the NGDSS knowledge base is enriched. An initial core body of knowledge may be manually curated by translating disease entries in a textbook into structured patterns of diseases (Figure 4, panel C provides an example [42]).

### Prioritizing Possible Diagnoses

Considerations beyond the prior probability of potential diagnoses on the differential diagnosis list come into play when making clinical decisions on investigation and treatment. The most probable disease may be of little practical importance to the patient’s outcome. On the other hand, missing the diagnosis of a severe, albeit less-likely, disease on the differential diagnosis may have grave consequences. Thus, the test and test-treat thresholds [43] may vary by disease severity. Seriousness may be reflected by measurable factors such as survival rate, complication rate, quality of life, and impact on productivity. Scoring for disease severity can be used to help in prioritizing the use of diagnostic tests and treatment modalities.

Likewise, the degree of urgency of conditions on the differential diagnosis list also has practical implications. Some conditions are considered medical emergencies (eg, stroke, malignant hypertension, or myocardial infarction) and require immediate measures to be taken by the physician, whereas in others the course and outcome are not changed by delaying treatment. In presenting information to the user, an NGDSS may indicate the need to act fast when such conditions are considered.

### Distinguishing Between Conditions on the Differential Diagnosis List

NGDSSs should provide next-step advice to optimize the diagnostic workup. Listing questions that, if answered, could narrow the differential diagnosis can be useful. Performance measures of diagnostic tests, contraindications for their use, and complication rates could be incorporated in their knowledge bases [44]. This information can be applied in the case of an individual patient to simulate a posttest probability given a prior probability and test result, such as using likelihood ratios [45]. Local factors such as test availability, costs, and medical insurance coverage can be considered to adapt generic recommendations to available resources. In the face of high rates of overuse and misuse of diagnostic tests [46], NGDSSs could reduce patient harm and costs by optimizing the diagnostic workup.

### Alignment With Workflow

Experience with current DSSs shows that their use is hindered by poor alignment with the clinical workflow. Most DSSs require at least some degree of redundant input of clinical information. NGDSSs must seamlessly integrate with EHR systems. Cognitive computing approaches can facilitate the interaction of physicians with NGDSSs. For example, a structured pattern may be interactively created using graphic user interfaces and touch screens (see Textbox 1 and Figure 4 for a suggested practical approach).

**Computer-aided diagnosis using a 3-step human-computer interactive process to capture structured patterned patient and disease representation.**

1. *On-the-fly, user-supervised, automated term extraction from clinical notes:* As a physician is typing in clinical notes, a real-time list of natural language processing-extracted medical terms is generated by mapping notes to standard controlled terminology. Terms are then reviewed by the physician, who can check those that apply to the patient or select keywords from the text and quickly add them to the term list.

2. *Interactive creation of a structured “pattern” by connecting related terms*: With the advent of capacitive touch screens, dragging, pinching, and swiping have become intuitive to anyone using a mobile phone. A structured patient timeline can be generated by the physician by arranging the extracted keywords on a time axis. This process would turn a unidimensional list of terms into a 2-dimensional pattern. Much like temporal relations, semantic relations between terms could be annotated by the physician. Among those could be cause-and-effect relations, degree of certainty, or contradicting evidence. Graphic determinants such as shape, size, color, or transparency may be used to distinguish between event types or represent symptom severity. Physician time constraints will limit the granularity and richness of the patterns created. Nevertheless, even a structured patterned sketch of a patient’s presentation can carry more information than a list of findings.

3. *Pattern enhancement using automatically extracted patient data*. The structured presentation sketch can serve as an anchor for structured data automatically extracted from the electronic health record to enhance the pattern. Such data types may include vital signs, laboratory results, keywords from imaging and pathology reports, and background medical information (eg, comorbidities).

A Bayesian network could be continuously trained to match a new patient pattern on a large set of existing patterns, and to rank the diagnoses to which similar patterns are attributed by their prior probability.

### Limitations, Challenges and Potential Solutions

Implementation of the proposed approach for NGDSSs requires major health care stakeholders to make substantial, prolonged, and coordinated efforts. To bring NGDSSs to life, major technical and regulatory challenges will have to be met. Here, we mention some of the barriers NGDSSs face and propose ways to overcome them.

#### Crowdsourcing Data Quality

The medical domain is characterized by tight regulation of knowledge to assure quality. In this sense, crowdsourcing is an unorthodox approach. While offering access to much more knowledge than is possible using traditional methods, crowdsourcing carries an obvious risk of collecting unreliable information. Labeling structured patterns with the diagnosis subsequently made would be accurate in some cases; however, with misdiagnosis being not uncommon [7], some labels will undoubtedly be wrong. Like in other domains where big data is used, large numbers may provide protection against misinformation. Ongoing expert evaluation of outliers in the knowledge base can also help eliminate errors. Ways to select appropriate contributors will have to be sought. These may include proof of medical education, licensure, or affiliation with a recognized institution. Feedback from peers could be used to flag errors or unreliable contributions. Contributor evaluation could be automated, for instance, by measuring the degree of similarity of patterns generated by a user to patterns with the same label generated by other contributors. If similarity is consistently low, the reliability of that contributor would be questionable. To build the initial, core knowledge base, textbook disease entries could be translated into a structured format, for instance, with the help of medical students as part of their training. New patterns will be compared with this knowledge base and inconsistencies found could be manually reviewed. NGDSSs could be evaluated in controlled settings, such as by presenting them with standard cases (as has been done with current DSSs), or comparing their performance with that of physicians in real-life settings. The Human Diagnosis Project [32] model, in which cases are reevaluated by clinicians, can be useful as a quality control tool.

#### Data Sharing

Real-time sharing of structured patterns would not be possible unless authorities and other stakeholders are convinced that patient privacy is protected. The use of structured patterns eliminates the need to manually deidentify clinical notes and may facilitate sharing. However, the use of many different formats for presenting clinical data will require efforts to align EHR data from various products. The widely used Observational Medical Outcomes Partnership common data model [47], as implemented in the Observational Health Data Sciences and Informatics project [48], is a promising approach to this challenge. A central knowledge base may shorten the turnover time to provide answers to users, but medical institutions may choose to store their data on local servers, which will need to support application programming interface allowing controlled interrogation of their data.

#### Complex Presentations

Medical students are encouraged to find a single disease that would explain a patient’s symptoms and signs. With an ageing population, comorbidities and polypharmacy are common. Comorbid conditions and medications used to treat them may alter the manifestations of a disease; the interplay between multiple factors with potential bearing on the clinical picture may make it impossible to attribute a pattern to a single etiology. This is true for an NGDSS but also, in many cases, for clinicians as well. For example, shortness of breath in the context of a respiratory tract infection may be caused by pneumonia, but in a patient with known heart failure, decompensation with pulmonary congestion may also explain the symptoms. Data extracted from the EHR of a patient and anchored to his or her structured pattern may help gain better understanding of the clinical picture. For instance, a patient’s problem list and past laboratory results may put current findings in the right context. Unfortunately, variable quality of EHR data and mixture of clinical and billing information may limit the degree to which uncertainty could be reduced. New tests enhance our knowledge but may bring instances where current medical practice is simplistic to the surface. For instance, in a recent retrospective analysis, almost 5% of patients with a molecular diagnosis had 2 to 4 diagnosis accounting for their phenotype [49]. This is a challenge for humans and NGDSSs alike; however, NGDSSs are better positioned to handle genomic data (as well as other high-volume data types) and learn genotype-phenotype associations.

#### Alignment With Workflow

Admittedly, at least in the foreseeable future, even the most user-friendly NGDSSs would require clinicians to invest time in acquainting themselves with their use and interacting with them in the clinical setting. This is a challenge for work-overloaded physicians, many of whom do not trust DSSs. Attempts to structure history taking (eg, [50]) have not gained popularity due to usability issues. Thus, for an NGDSS to be used, it has to minimize interaction time. Even more importantly, NGDSSs will have to reward users by providing useful insights. One way to give clinicians back the time invested in interacting with an NGDSS would be to automatically translate a user-generated structured pattern into narrative notes. Apart from generating a differential diagnosis list, presenting an extract of the evidence used by the system when reaching its conclusions, as well as links to source documents, could make the system’s conclusions more understandable to physicians and increase their trust. The direct feedback an NGDSS could provide can serve as a powerful tool in developing clinical expertise and may be a strong driving force for clinicians to use it. Using NGDSSs may also save time by eliminating the need to run a Web search or look for relevant evidence in other sources (eg, UpToDate, textbooks). Nevertheless, due to the extra effort required to use NGDSSs, more obscure or difficult cases are likely to be overrepresented in their knowledge base. Ways to correct for this when estimating prevalence will have to be developed.

## A Learning Health Care System

NGDSSs can realize the vision of Ledley and Lusted [31] for a learning health care system through knowledge reuse. We describe some of the potential benefits of this approach in this section (Figure 3).

### Better Characterization of Diseases and Syndromes

There are probably as-yet unidentified diseases and syndromes. Some syndromes go unnoticed due to their rarity, and those could be identified through analysis of very large datasets. A structured clinical pattern can serve as an anchor for all patient-related structured data (eg, laboratory results, imaging tests) in a patient record. The result would be a rich representation of the manifestations of the patient’s disease(s), in which laboratory and imaging results are put in clinical context. This can help us understand accumulating genetic, proteomic, and microbiomic information and its association with clinical disease at the individual patient level. Integrated knowledge can help break down syndromes (eg, systemic lupus erythematosus and inflammatory bowel disease) to their underlying causes. Cohorting patients with similar structured patterns could potentially support more accurate outcome prediction and more reliable detection of adverse reactions to medications and other interventions.

### Improved Medical Education

Apprenticeship is a major pillar in the training of clinicians, appreciating that effective learning takes place through practice and direct, immediate feedback [51]. Whereas clinicians do receive feedback on their decisions through following up on their patients, minimizing trial-and-error-based learning is advisable. An attending physician provides feedback to his or her residents during rounds. However, most of the medical work is done between attending visits and does not involve high-quality feedback. An efficient and reliable NGDSS could serve as a mentor to both practicing physicians and medical students. As offered by current DSSs, NGDSSs could generate patient vignettes used for problem-based learning and diagnostic performance evaluation.

### Enhanced Disease Surveillance Powered By Real-Time Clinical Data

Users’ Internet activity has been shown to detect disease outbreaks before regulatory agencies can detect them [52,53]. Likewise, when NGDSSs are used, real-time clinical data may enhance early outbreak detection with a higher signal to noise ratio. Early detection is key to containing an outbreak, since by the time the first cases are identified, other individuals have likely been infected and may be spreading the disease. Enhancing the ability to detect clusters of unusual cases seen in the clinical setting in real time, even before a clinical or laboratory diagnosis has been established, can enable taking control measures earlier.

### Improved Health Care in Limited-Resource Settings

Populations living in limited-resource settings are typically underrepresented in published medical reports. Cultural issues, limited availability of health professionals and diagnostic tests, and other factors may influence the ways diseases are first encountered and diagnosed by clinicians in such settings. Indeed, diagnostic errors in primary care are more common in low- and medium-income countries [54]. NGDSSs could help bypass the publication bias by collecting, analyzing, and sharing locally relevant knowledge. Global knowledge collection is particularly important in the age of global travel. Returning travelers can present with diseases acquired during travel that are rarely encountered in their homeland. Comparing their presentation patterns with patterns commonly seen in the places they had visited could help local physicians overcome knowledge gaps and the availability bias.

### Conclusion

Computer-aided diagnosis has for decades been the Holy Grail of medical informaticians. The extreme complexity of constructing an efficient and sustainable system is reflected by the infrequent clinical use of DSSs despite the vast efforts that have been put into developing them.

On the one hand is an expanding domain knowledge, increasingly complex patients, and a high burden of diagnostic errors. On the other, EHR systems have become ubiquitous; powerful computers enable sophisticated analytics; the Internet can connect physicians from around the globe in real time; and human-computer interaction technologies have ripened. Taken together, there is both a real need for NGDSSs and the technology to meet it. We are laying a conceptual framework for developing NGDSSs that relies on structuring clinical notes; real-time sharing of patient structured patterns; democratization of knowledge generation, maintenance, and reuse; and integration of epidemiologic data to support the complicated task of making a diagnosis.

Development of NGDSSs will be very demanding, yet we argue that their potential utility justifies the investment required to realize them. The future of computer-aided medical diagnosis lies ahead and will likely change the way medicine is practiced.

## Abbreviations

DSS: diagnosis support system
EHR: electronic health record
NGDSS: next-generation diagnosis support system
